# Late-onset leukoencephalopathy with cerebral calcifications and cysts: case report and review of the literature

**DOI:** 10.1186/s12883-016-0543-1

**Published:** 2016-02-06

**Authors:** C. Stephani, S. Pfeifenbring, A. Mohr, C. Stadelmann

**Affiliations:** Department of Clinical Neurophysiology, University Medical Center Göttingen, Robert-Koch-Straße 40, 37075 Göttingen, Germany; Department of Neuropathology, University Medical Center Göttingen, Robert-Koch-Straße 40, 37075 Göttingen, Germany; Department of Neuroradiology, University Medical Center Göttingen, Robert-Koch-Straße 40, 37075 Göttingen, Germany

**Keywords:** Leukoencephalopathy, Calcifications, Cysts, Glucocorticoids

## Abstract

**Background:**

Leukoencephalopathy with calcifications and cysts (LCC or Labrune disease) is a relatively recently defined and exceptionally rare disease in which parenchymal cysts and calcifications within a widespread leukoencephalopathy can cause a broad spectrum of neurological symptoms. The cause of the disease is unknown. Manifestation is usually in childhood or adolescence, while onset in adulthood has been described in 19 cases.

**Case presentation:**

Here we report a case of an adult-onset LCC of a Caucasian woman who became symptomatic at age 70 as confirmed by typical neuroimaging and neuropathological findings. After resection of left mesioparietal space-occupying cystic brain tissue the patient has so far remained clinically stable during one year of follow-up with a continuous treatment with glucocorticosteroids.

**Conclusion:**

To our knowledge this report of a patient who became symptomatic at age 70 represents the oldest age-at-onset case of LCC described so far.

## Background

Leukoencephalopathy with cerebral calcifications and cysts (LCC) is a leukoencephalopathy syndrome defined some 20 years ago [1]. Its name contains the triad of its disease-defining features. Having been first described in 1996 by Labrune et al. [1], the disease which sometimes bears his name, has been reported in fewer than 50 cases in the literature as of 2014 [2, 3]. Disease onset takes place in childhood-adolescence as well as in adulthood; while the course of the disease has been described as more aggressive in childhood, the main findings apparently are independent of age. Histopathology reveals ectasia of small vessels, calcification of the parenchyma as well as gliosis. Occurrence of “Rosenthal-fibers” – hematoxylin and eosin stain-positive aggregates of intermediate filaments – is regarded as a particular feature. Given its very specific characteristics, the diagnosis of this syndrome may be established through neuroimaging alone. In the largest series of 15, mainly childhood-onset, patients with LCC, computer tomography (CT) in all patients revealed parenchymal calcifications of varying degrees, typically involving the basal ganglia and the thalamus. Half of the patients also showed calcifications of the dentate nucleus and brainstem [3]. In this same cohort, magnetic resonance imaging (MRI) showed periventricular and deep white matter leukoencephalopathy that was symmetrical in all but two patients. According to this report, cysts were found in all brain areas with no clear preference for any region. The clinical presentation of the patients thus depends on the extent and location of the brain lesions which often involve extended edema, and includes all types of central neurological findings. The disease may be fatal within several years after diagnosis, most often due to intracerebral bleeding, but may also remain stable for years. Regarding the etiology, a recent report on two sisters with histopathologically confirmed LCC indicates that this is a genetic and possibly autosomal recessive disorder [4]. Here, we present another case of an adult-onset patient with histopathologically confirmed LCC, in which characteristic findings are described and set in relation to the available literature.

## Case presentation

A 70-year-old right-handed woman had complained about dizziness and unsteadiness of gait for several months. She also reported moderate headaches which she had not had previously. Moreover, she experienced memory deficits as well as difficulty finding words. She also noted that while experienced in target shooting with a pistol, her accuracy had decreased significantly within this same period. Aside from arterial hypertension and a deep vein thrombosis that had occurred within the year prior to admission, there were no major co-morbidities. The family history for neurological disorders was reported as normal. However, the patient’s parents were deceased and the patient did not have siblings.

**Fig. 1:**
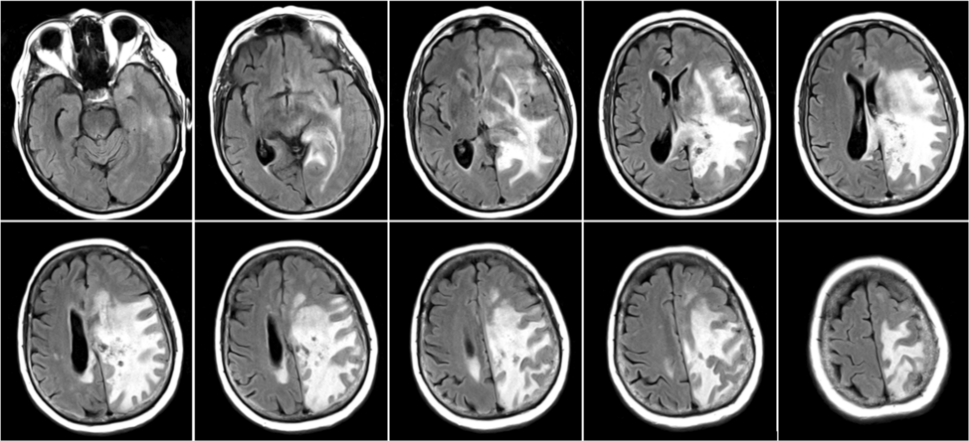
Fluid-attenuated-inversion recovery (FLAIR) sequence of the initial MRI

**Fig. 2:**
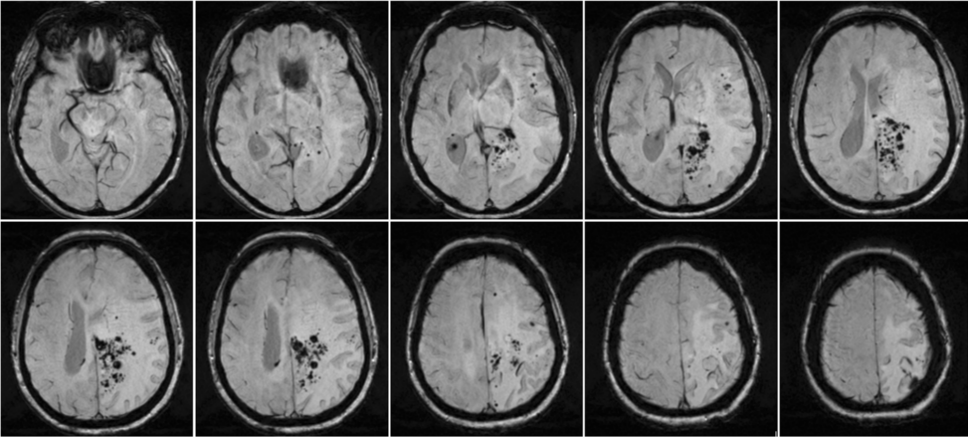
Susceptibility-weighed (SW) sequence of the initial MRI

A first MRI-scan revealed extended brain edema of the left hemisphere including left parietal cystic lesions. A brain biopsy was performed and repeated several months later. Both brain specimens obtained contained spongiform tissue with microangiopathy and no signs of malignancy, but did not allow a firm diagnosis to be made. The patient was then admitted to our hospital. Upon examination we found an incomplete right-sided hemianopia and very discrete right-sided hemiparesis, including a reflex difference with more definite reflexes on the right side. Coordination was mildly impaired. Additionally, she demonstrated word-finding difficulties and signs of apraxia of the right hand. She had been on high-dose glucocorticosteroids initiated probably after her first MRI; in the meantime she had also received medication against arterial hypertonia and antiepileptic medication following a first epileptic seizure. A recurrent MRI confirmed the initial findings of mainly left hemispheric leukoencephalopathy and a left parietal cyst (Figs. 1 and 2).

**Fig. 3:**
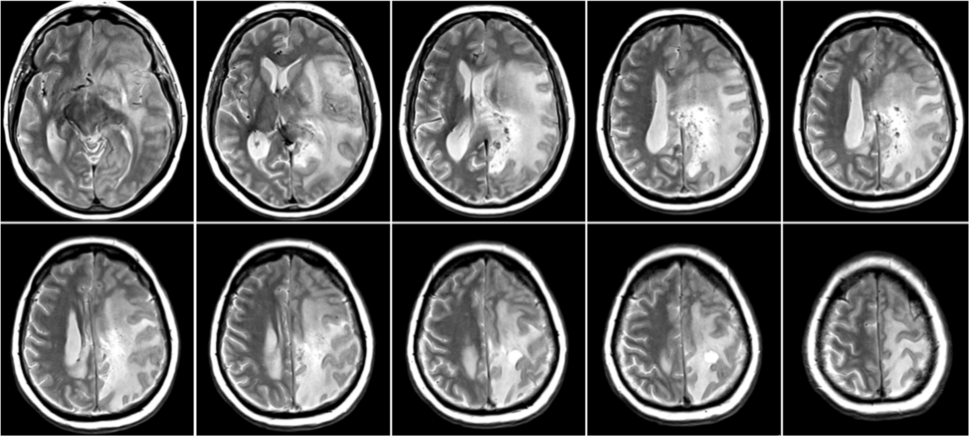
T2-weighted MRI 6 weeks after the initial MRI and after tapering off glucocorticoids. Note the more pronounced midline shift to the right as compared to the prior MRI

After reviewing the results of the first two biopsies that had been performed while the patient was already taking glucocorticoids, we decided to discontinue the steroid treatment in order to improve the diagnostic accuracy with respect to the exclusion of a cerebral lymphoma. When the patient was re-admitted 4 weeks later, she reported a worsening of her symptoms, her foremost complaint being memory problems. Consistent with this, the patient only scored 10 of 30 possible points on the mini-mental-status-examination (MMST), while during the first admission her test performance had been 21 of 30 points. Radiologically, the left hemispheric brain edema had increased resulting in a more pronounced midline shift of the left hemisphere than had already been present in the first imaging (Fig. 3). CCT showed evidence of some intraparenchymal calcification, but none in the basal ganglia, thalamus or cerebellum.

**Fig. 4:**
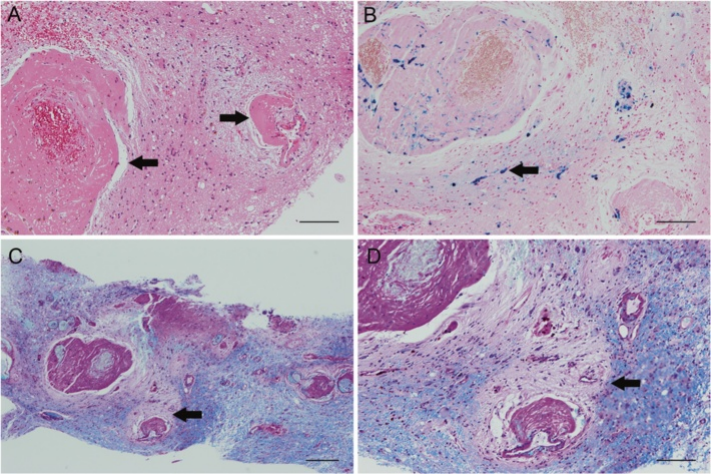
Typical histopathological findings. Arrows indicate the respective specific features. **a** Blood vessels with hyaline degeneration. **b** Signs of micro-bleedings. **c** and **d** Perivascular loss of myelin (LFB/PAS staining) (scale bars a, b, d = 100 μm, c = 200 μm)

A resection in the left mesioparietal region with an area of dense vascular anomalies was performed after 2 more weeks. Typical findings of the biopsies and resection are shown in Fig. 4. These showed blood vessels with hyaline degeneration, signs of micro-bleedings and perivascular loss of myelin. Even though the typical mineralizations and cysts were not detected within the tissue the findings in conjunction with the results of the MRIs were regarded as suggestive for a leukoencephalopathy with calcifications and cysts.

**Fig. 5:**
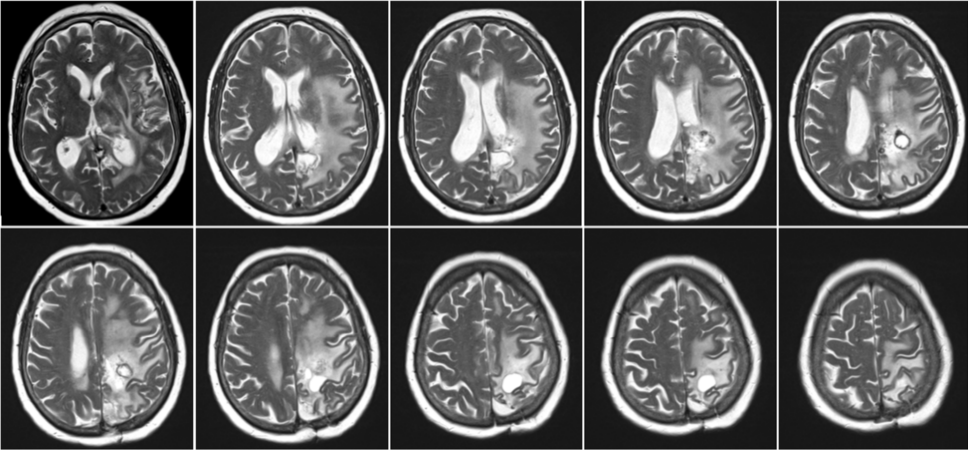
T2-weighted MRI one year after initial presentation and 10 months after left mesioparietal resection. Increase in size of the left posterior cyst as well as periventricular intracystic bleeding. Stable course of the leukoencephalopathy

Symptoms did decrease postoperatively and after resumption of the corticosteroids. However, after 2 months corticosteroids were once more tapered off and the symptoms again became more aggravated. Medication was therefore re-established as a long-term treatment with glucocorticoids and continues to date. The MRI one year after the initial presentation at our institution did show a stable course regarding the leukoencephalopathy which had even rather mildly decreased. Nevertheless, a small increase in the size of the largest cyst in the left posterior quadrant could be observed, as well as slight bleeding in one of the smaller cysts anterior to it (Fig. 5). Unfortunately, in the most recent follow-up visit 6 months later, the patient, though is still under constant treatment with corticosteroids, showed marked clinical deterioration; she was only able to communicate due to a mildly fluctuating sensory aphasia and completely relied on her husband’s assistance. Despite this clinical progression the MRI did not show significant changes. Her anti-epileptic medication was increased but did not profoundly change the clinical patient’s presentation.

Medication-wise, upon prolonged treatment with corticosteroids in doses of 2–4 mg fludrocortisone, the patient not unexpectedly developed typical signs of Cushing-syndrome, including weight gain, skin fragility and superficial skin infections. While the patient initially had received anti-coagulation treatment with phenprocoumon due to a deep vein thrombosis, this was discontinued in view of the risk of intracerebral bleeding with this diagnosis.

Ophthalmological examination confirmed a normal fundoscopy. The CSF revealed a mild elevation in the total protein (721 mg/l), but no cells, normal lactate and no oligoclonal bands. Rheumaserology and serology of vasculitis-typical antibodies was unremarkable. Results of neurophysiological testing accorded well with the clinical symptoms and neuroimaging, and showed centrally increased latencies of the sensory-evoked potentials involving the left hemisphere, as well as a marked slowing of the EEG rhythms generated by the left hemisphere with left temporal epileptiform activity in the EEG. This patient additionally received several modalities of neuroimaging including regular MRIs, a fluor-deoxy-glucose positrone-emission-tomography (FDG-PET), a single-photon-emission-computer-tomography (SPECT) as well as an angiography. The cerebral angiography revealed an unspecific enhancement of vascular contrast with early venous drainage in the left parietal region resembling small shunts. There was otherwise no abnormality of the brain-feeding arteries and no sign of vasculitis. FDG-PET and SPECT showed concordantly more left than right parieto-occipital hypometabolism and hypoperfusion.

### Review of the literature

Upon a search of the literature in the Medline database, we identified 19 more cases of adult-onset LCC, the data of which were available to us. One of these cases was published as part of a series of 15 mainly juvenile-onset LCC. However, the clinical data of that specific case were not fully accessible within that publication. We were therefore able to include this case in this review [3]. In another case report, the diagnosis of LCC was established at age 42, but long-standing dystonia and spasticity since early childhood did not allow inclusion of this case into the adult-onset case series [5]. Including our case, we found the following characteristics based on a total of now 20 published cases of 10 male and 10 female patients. Diagnostically, histopathology was available in 90 % (18/20) and CSF in 35 % (7/20) of the patients. Seizures were part of the recent or overall medical history in 40 % (8/20). Therapeutically, in 60 % (12/20) a neurosurgical resection was performed. The prescription of steroids was reported in 20 % (4/20) (Table 1).

**Table 1:** Adult-onset cases of leukoencephalopathy with calcifications and cysts published until end of 2014

Age	Sex	CSF	CCT	MRI	Histology	Neurological symptoms	Seizures	Therapy	Source
18	F	–	Bilateral foci of calcification in white matter, both thalami and left basal ganglia, cysts in right frontal and left temporal lobes.	Right frontal and left temporal lobes cysts with thin uniformly enhancing walls. Increased T2 signal bilaterally in the white matter with sparing of the gray matter. No cerebral atrophy.	Extensive gliosis with Rosenthal fibers, rarefied microcystic foci, and a larger cyst with abundant hemosiderin along the wall. Extensive calcifications and absence of any abnormal blood vessels.	Seizures, learning disability	Y	–	Ogles et al. 2014 [4]
19	M	–	Low-density cysts with high-density ring and bilateral calcifications.	Extensive leukoencephalopathy. Numerous bilateral cysts with hyperintense boundaries (T1 + T2) of various sizes including thalamus, basal ganglia and left ventricle. Cyst wall enhancement. Heterointense cystic content in T1 and FLAIR.	Pronounced reactive gliosis with Rosenthal-fibers. In addition, focal hemosiderin deposits, which indicate previous hemorrhage, and microcalcifications. Many ectatic vessels and angiomatous changes with cellulose-like degeneration. Analysis of the cyst fluid did not suggest malignancy or infection.	Weakness of right limbs; vision loss	N	Excision of left frontal cyst	Wang et al. 2013 [13]
19	M	–	Calcifications in pons, basal ganglia, and all lobes mostly in white matter. Some with cysts. Diameters from a few to 15 mm. Ring enhancement of the cyst wall.	Many cysts associated with calcifications, increased signal intensity in the bulbous, pons, bilateral cerebellar, and cerebral peduncles, basal ganglia, white matter, not in cortical gray matter. Vasogenic edema. Contrast-enhanced scans showed ring enhancement in all cysts.	Angiomatous changes, numerous small, tortuous blood vessels, either contiguous or separated by nervous tissue, hyaline vascular thickening with fragmented rough reticulin fibers and associated irregular calcifications. Microhemorrhagia, ferric iron deposits Rosenthal fibers surrounding the abnormal vascular configurations.	Seizures	Yes	Diagnostic right temporal lesionectomy; after 2 years removal of a pontine cyst	Sener et al. 2006 [14]
24	M	–	Dense calcifications in left corona radiata and bilateral thalamus; pontine cyst; Ventriculo-megaly.	Additionally to CCT, peri-ventricular and pericyst fluid-attenuated inversion recovery (FLAIR) signal abnormalities.	Analysis of the green cystic fluid and the thickened cystic wall: no evidence of neoplastic processes. No angiomatous rearrangement of microvasculature or Rosenthal fibers.	Several weeks of progressive headache, imbalance and diplopia Exam: right internuclear ophthalmoplegia (INO), hypesthesia in left arm.	–	1. bilateral suboccipital craniectomy and cyst drainage 2. cyst draining shunt	Berry-Candelario et al. 2011 [10]
25	F	–	Bifrontal cysts and dense calcification in both thalami and left basal ganglia; left frontal cyst with a hemorrhage.	Bifrontal cysts with thin uniformly enhancing walls and also extensive bilateral white matter increased T2 signal with sparing of the gray matter. No cerebral atrophy.	Extensive gliosis with Rosenthal fibers, abnormal thick-walled congo-red negative and acid-Schiff positive blood vessels, rarefied microcystic foci, and a larger cyst with abundant hemosiderin along the wall.	Left hemiparesis postpartum and 1 week of headache	N	Resection of a cyst	Ogles et al. 2014 [4]
26	M	Normal protein + glucose, slightly increased pyruvate	Bilateral symmetric calcifications at basal ganglia, thalamus, cerebellum, triventricular hydrocephalus, cerebral edema.	Additionally, cystic peripherally enhancing cerebellar and thalamic masses on the left side; hypointense on T1 and hyperintense on T2; calcified focus near cyst.	Calcification of the vasculature, areas of demyelination, and gliosis. No signs of malignancy.	Vertigo, vomiting, imbalance. Exam: Severe disorientation, bilateral nystagmus, papilledema, right-sided clonus, gait ataxia, Babinski positive.	–	–	Daglioglu et al. 2009 [15]
27	F	–	Nodules in the basal ganglia, thalami and centrum semiovale, densely calcified at CT	Large left thalamic cyst compressing aqueduct and third ventricle with triventricular hydrocephalus cerebellar cyst; cerebral and left cerebellar white matter hyperintensities on T2; post contrast enhancing nodules in the basal ganglia, thalami and centrum semiovale.	Angiomatous-like microangiopathy, calcifications in brain tissue and vessel walls, and abundant Rosenthal-fibres with predominant perivascular arrangement.	Initially: Symptoms of increased brain pressure. 6 months after surgery: slight dysarthria, mild left IVth nerve palsy, right mydriasis, lower limb incoordination, left pyramidal signs. Mild dysexecutive impairment.	-	3^rd^ ventri-culostomy and removal of cerebellar lesion	Marelli et al. 2008 [16]
30	F	Normal	Calcifications in right thalamus, left basal ganglia, right cerebellum, with cystic formation on right parietal lobe. Asymmetric diffuse leucopathy.	Acute ischemic lesion in posterior left internal capsule. Asymmetric white-matter hypersignals, always surrounding cysts and calcification foci on T2. Gradient echo imaging revealed extent of calcifications, which were not seen on CT or conventional MRI. Ring-contrast enhancement of cysts walls and adjacent to calcifications. MRI-angiography was normal.	–	Right-sided hemiparesis	N	–	Wargon et al. 2008 [17]
30	M	–	–	Expansive solid/cystic interhemispheric lesion with hemosiderin deposition and high capillary density (MR perfusion) in its walls. Multiple enhancing nodular lesions in supra- and infratentorial brain parenchyma some with hemorrhagic component on T2. Symmetric calcifications in periventricular white matter, basal ganglia, brainstem, dentate nucleus.	Inconclusive	Headache for 7 days	N	Surgery	Bertotti et al. 2011 [18]
31	M	–	Bilateral calcification in the basal ganglia.	Extensive bilateral leukodystrophy and cysts with marginal enhancement after intravenous contrast; progression in repeat MRI.	Foci of dystrophic calcifications and focal accumulations of macrophages xanthomized. No signs of malignancy.	Tonic-clonic seizures for 7 years	Y	5 surgeries for removal of brain cysts	Pessoa et al. 2012 [19]
36	M	–	Extensive calcification in basal ganglia, deep cerebellar nuclei, right thalamus.	Diffuse symmetric white matter hyperintensity; large rounded cystic lesion in the left occipito-temporal region.	–	Mild right hemiparesis, Tremor, seizures for 5 years; Exam: strength 4/5 right; mild spasticity	Yes	Anti-epileptic with Valproate	Gulati et al. 2011 [20]
42	M	–	Left parietal ring -enhancing lesion with calcification.	Multiple, irregular, and heterogeneously enhancing lesions scattered throughout the brain.	Multifocal vascular proliferation associated with necrosis, gliosis, and macrophage infiltration.	Right footdrop for 2 weeks	No	Corticosteroids	Kleinschmidt-Demasters et al.2009 [21]
44	F	–	Calcifications in the basal ganglia.	Diffuse cerebral and cerebellar leukoencephalopathy, mass effect with right-to-left shift, with cysts and enhancing lesions in the cerebral white matter. Type I Chiari malformation, cervical syrinx.	3 brain biopsies between 1999–2003.	Generalized daily headaches; Ataxia, seizures, mild cognitive dysfunction over last 6 years	Yes	Cortico-steroids ➜ responsive-ness	Kleinschmidt-Demasters et al. 2009; Corboy et al. 2006 and [8, 21]
45	M	Normal	Asymmetric basal ganglia calcifications. Small right calcification on skull X-ray at age 12 years.	Diffuse leucoencephalopathy and multiple cysts with a rim of post contrast enhancement. Normal spinal MRI.	Dilated blood vessels with a thickened wall, arranged in an angiomatous-like fashion, associated with abundant microcalcifications and Rosenthal fibers.	Progressive gait impairment for 3 years Exam: atactic-spastic gait	Y (2 GTC at age 12 + 18)	Resection of the right frontal cyst	Marelli et al. 2008 [16]
50	M	Slightly xantho-chromic; 26 leuko-cytes, protein 1060 mg/l	Multiple calcifications in basal ganglia and white matter.	Multiple bilateral cerebral cystic lesions and large right cerebellar cyst with mass effect; diffuse T2 hyperintensity.	Pilocytic gliosis with Rosenthal fibers, prominent angiomatous changes, microcalcifications, microhemorrhages and hemosiderin pigment deposits. Small blood-filled vessels, many showing hyalinized walls. Extensive Microcalcifications + concentric fine calcification around blood vessel walls.	Headache for 4 months, progressive unsteadiness of gait for 1 month, recurrent vomiting for 1 week Exam: papilledema, finger-nose and heel-knee dyscoordination, dysdiadochokinesia; spastic-atactic gait	Dur-ing child-hood as-soci-ated with fever	Posterior fossa- crani-ectomy with resection of a cyst	Ummer et al. 2010 [22]
55	F	Normal	Abnormalities	Widespread leukoencephalopathy more prominent anteriorly with multifocal cysts up to 1 cm in diameter. Numerous punctate-enhancing lesions distributed throughout the cerebrum. Little mass effect and edema.	Angiomatous small blood vessels with relatively thin walls and minimal hyalinization but with focal fibrinoid vascular necrosis and microhemorrhages; numerous Rosenthal fibers.	None (incidental finding after car accident)	No	Corticosteroids	Kleinschmidt-Demasters et al.2009 [21]
59	F	Protein: 890 mg/l No cells No OCBs	–	Diffuse infra- and supratentorial leukencephalopathy, extensive calcifications in basal ganglia, thalamus, cerebellum (dentate nuclei), multiple voluminous cysts.	Pronounced reactive gliosis with numerous Rosenthal fibers, mild myelin pallor without signs of active demyelination. Focal hemosiderin deposits and microcalcifications.	Increasing urinary urgency for 5 years, change in behavior with increasing apathy for 1 year; left homonymous hemianopia	–	Resection of a cyst	Kaffenberger et al. 2009 [9]
65	F	–	Calcifications in the cerebellum and basal ganglia, frontoparietal ICH with 1-cm midline shift + extensive white matter hypodensities. On admission as well as 6 years prior.	Increased FLAIR around hemorrhage and in the bilateral periventricular white matter. T2 hyper-intensity, consistent with cysts, posterior to the left frontal horn and in the left cerebellum. Follow up MRI after operation demonstrated resolution of the hemorrhage and surrounding edema.	Gliosis and thickened blood vessels, but no amyloid deposition or malignancy.	Worsening of residual hemiparesis on the left and headache for 3 weeks Exam: left hemineglect, spastic left hemiparesis, confusion	N	Right hemi-craniectomy and resection for the hemor-rhagic mass	Banks et al. 2013 [6]
69	F	–	Calcifications of thalamus, basal ganglia, deep white matter, brain stem and dentate gyrus.	Extensive white matter hyperintensities in T2 and FLAIR.	Organizing hemorrhagic tissue with gliosis, no angiomatous or hyalinized blood vessels, myelin loss, calicifications, Rosenthal fibers.	Confusion, ataxia, gait unsteadiness, repetitive falls, left homonymous anopia over several months	N	–	Coeytaux et al. 2011 [7]
71	F	total protein (721 mg/l), no cells, normal lactate	Some intraparenchymal calcification, but none in the basal ganglia, thalamus or cerebellum.	Extended brain edema of the left hemisphere including left parietal cystic lesions.	Blood vessels with hyaline degeneration, signs of micro-bleedings and perivascular loss of myelin.	Incomplete right-sided hemianopia, discrete right-sided hemiparesis,. Coordination mildly impaired. Word-finding difficulties and signs of apraxia	Y	Surgery + Corticosteroids	Stephani et al. [this report]

### Discussion

We describe the case of a 71-year-old female diagnosed with LCC and an onset of symptoms later in life than any so far reported in the literature. While there exists a previous report of another 71-year-old female with the same diagnosis [6] in that case the onset of symptoms was 6 years prior to diagnosis. Our patient however presented with first symptoms only a few months before a final diagnosis of LCC was made; rendering her the oldest patient to manifest first clinical LCC symptoms described so far. Here, the leukoencephalopathy was mainly left hemispheric. This is unusual, even though asymmetric distribution of this finding has been described before and may represent an early stage or varying expression of the disease [3]. In our case we observe typical cysts, these being a defining feature of the disease that have been reported in all previous cases. Also, the cysts showed dynamic changes in the course of the follow-up, which is also regarded as typical for LCC [3]. However, in contrast to previous descriptions of LCC, we found mild but not typically localized calcifications in the CCT. Indeed, most of the available case reports describe a rather typical neuroimaging that often included bilateral and marked calcifications within the basal ganglia, thalamus and cerebellum. While this pattern of parenchymal calcification resembles the pattern seen in Fahr’s disease, the differential diagnosis for Fahr’s disease is not accompanied by any of the additional features of LCC. We may thus speculate that the extent of LCC calcification is variable, and that the pattern we observe is also consistent with LCC.

The natural course of LCC appears to vary widely. Ages of clinical onset ranging from the first year of life to 70 years of age (including our case) have been reported, with some cases taking a fatal course and others maintaining clinical stability for years. Especially with respect to adult cases, it is not clear when the changes in cerebral structural occur. In one case, a 69-year-old Caucasian women presented with confusion, ataxia and left-sided hemianopia that had developed over the preceding several months, and the typical triad of LCC was subsequently confirmed through neuroimaging. However, neuroimaging that had been performed after a concussion 14 years prior to manifestation of the above-mentioned symptoms had already revealed the presence of cerebral calcifications and leukoencephalopathy, despite the fact that the patient was reportedly asymptomatic at that time; she thus underwent no follow-up [7]. In another case report, brain images made 6 years before the patient’s main presentation and likely clinical manifestation were available and showed the same leukoencephalopathy as well as calcifications as the later brain images. However, the cysts that had likely induced the new symptoms had not been present on initial imaging [6]. It therefore appears likely that − at least regarding adult-onset patients − there may be a long latency between structural and clinical manifestation of the disease. On the other hand, the formation of cysts apparently represents a more dynamic part of the disease with particular relevance for the clinical manifestation.

The etiology of the LCC is, as previously mentioned, unknown. A recently reported co-occurrence of LCC in two siblings strengthens the assumption that this is a genetically determined disease. Also a mutation of a gene encoding the cysteine protease inhibitor cystatin C (CST3) has been discussed as possibly relevant for the disease [6].

The pathogenesis of LCC apparently affects the structure and function of blood vessels, resulting in microangiopathy. It has been hypothesized that tissue hypoxia secondary to microangiopathy may then be responsible for the development of calcifications and cysts. Additionally, pathologic myelination may be responsible for the leukoencephalopathy. Especially, glial process thickenings similar to the Rosenthal fibers that are typical of LCC have been observed in Alexander’s disease, a demyelinating leukodystrophy associated with mutations in the glial fibrillary acidic protein (GFAP) [8]. However, the white matter changes seen on the MRIs of patients with LCC apparently do not represent demyelination, but are more likely secondary to brain edema, which would fit well with the increase and decrease in leukoencephalopathy observed with the respective inactive or active glucocorticoid treatment in our case. Moreover, previous clinical case reports have noted differences between the extensive MRI abnormalities with vast subcortical hyperintensities in the T2-weighted sequences resembling patterns seen in leukodystrophies and the comparably more benign clinical phenotype which may be regarded as an indicator of a MRI-signal abnormality of less severe significance than would be the case with widespread demyelination.

The most effective therapeutic approach would likely involve a combination of corticosteroid therapy and surgical procedures, depending on the extent of the mass lesion. As always, chronic corticosteroid treatment should be minimized to the smallest dosage capable of maintaining a therapeutic effect. Interestingly, in two case reports it is mentioned that patients who had previously suffered from frequent headaches improved with steroid treatment [8, 9]. This may or may not indicate that these steroid-responsive headaches are symptomatically associated with the structural brain anomalies of LCC. Additionally, anticoagulants should be avoided in patients with LCC since histopathology indicates abnormal microvasculature. Indeed, several case studies describe the occurrence of spontaneous intracranial bleeding, and one report mentions a fatal intracranial hemorrhage that occurred most likely as a side-effect of treatment with an oral anticoagulant for deep vein thrombosis [8]. Regarding invasive treatment, in most cases surgical procedures were usually performed to relieve the effect of the space-occupying cysts or lesions that often occur in LCC [10]. Cystic lesions especially in areas that are accessible to neurosurgery may be resected. Also, in less accessible areas fenestration and shunting have been described as therapeutic alternatives [10].

Regarding differential diagnoses, Coats plus syndrome has been identified as a syndrome closely related to LCC; it has been hypothesized that both syndromes may represent variants of a single entity. Although both syndromes feature leukoencephalopathy with calcifications and cysts, what distinguishes Coats plus syndrome from LCC is that the former also manifests with prominent retinal vascular changes and osteopenia. Since these features as well as other systemic signs are absent in LCC, it is currently classified as purely a brain disease. Also, Coats plus syndrome has recently been associated with mutations in the CTC1 gene that is related to telomeric function [11, 12]. The lack of a mutation in the CTC1-gene in cases of LCC and its manifestation only in the central nervous system are currently regarded as the main features distinguishing Coats plus syndrome from LCC.

## Conclusion

While onset of LCC most often is in childhood this disease can present late in life.

## Consent

Written informed consent was obtained from the patient for publication of this Case report and any accompanying images. A copy of the written consent is available for review by the Editor of this journal.

## Abbreviations

CCT: cerebral computer-tomography
CSF: cerebrospinal fluid
CT: computer tomography
FDG-PET: fluor-deoxy-glucose positrone-emission-tomography
LCC: leukodystrophy with calcifications and cysts
MMST: mini-mental-status-examination
MRI: mangetic resonance imaging
OCBs: oligoclonal bands
SPECT: single-photon-emission-computer-tomography
